# Novel immunoassays for TDP‐43 detection in plasma and CSF

**DOI:** 10.1002/alz70856_098094

**Published:** 2025-12-24

**Authors:** Catherine Demos, Nikhil Padmanabhan, Sree Uttarala, Leonid Dzantiev, James D Berry, Anu Mathew, Martin Stengelin, George Sigal, Jacob N Wohlstadter

**Affiliations:** ^1^ Meso Scale Diagnostics, LLC., Rockville, MD, USA; ^2^ Massachusetts General Hospital, Boston, MA, USA

## Abstract

**Background:**

TAR DNA‐binding protein‐43 (TDP‐43) has an important role in the pathogenesis of several neurodegenerative diseases including amyotrophic lateral sclerosis (ALS), frontotemporal dementia (FTD), Alzheimer's disease (AD), and limbic predominant age‐related TDP‐43 encephalopathy (LATE). Improved immunoassays for detecting TDP‐43 and its disease‐associated modifications are needed, based on the hypothesis that proteins involved in disease pathology can serve as effective biomarkers.

**Methods:**

Antibodies were screened against purified TDP‐43 proteins and fragments, brain lysate, cerebrospinal fluid (CSF) and plasma from individuals with ALS (provided by MGH) and healthy controls (commercially obtained) to develop and preliminarily evaluate research use only (RUO) standard and ultrasensitive S‐PLEX assays for TDP‐43 and pTDP‐43.

**Results:**

The full length TDP‐43 assay in standard format had a quantitative range of 10‐275,000 pg/mL and sufficient sensitivity to quantitate 100% of the tested plasma samples. The assay displayed excellent dilution linearity. Whole blood, plasma and platelet‐rich plasma show higher concentrations than red blood cells, serum and platelet‐poor plasma, suggesting a need for good control over efficiency and timing of the separation of plasma from platelets and blood cells to avoid pre‐analytical effects. To measure the lower TDP‐43 levels in CSF samples, the more sensitive S‐PLEX TDP‐43 assay was used. It had a quantitative range of 5‐34,000 pg/mL and was able to quantitate 48% of the tested CSF samples. The S‐PLEX pTDP‐43 assay was used to measure TDP‐43 phosphorylated at S409/410 in plasma samples. It had a quantitative range of 1‐50,000 pg/mL, and was able to quantitate 100% of the tested plasma samples. Specificity was confirmed by testing phosphorylated and non‐phosphorylated purified protein. To preliminarily evaluate the assays, a small set of plasma and CSF samples from individuals with ALS was tested. Relative to the control samples, the ALS samples had higher median plasma TDP‐43 levels (ratio = 4.8, *p* =  0.0018), lower median CSF TDP‐43 levels (ratio = 0.49, *p* =  0.01), and non‐significantly higher plasma pTDP‐43 levels (ratio = 1.9, *p* =  0.11).

**Conclusions:**

Newly developed immunoassays for TDP‐43 and pTDP‐43 provide powerful tools for research on neurodegeneration biomarkers.

